# Southern African HIV Clinicians Society guideline on pre-exposure prophylaxis to prevent HIV

**DOI:** 10.4102/sajhivmed.v26i1.1713

**Published:** 2025-04-11

**Authors:** Pippa Macdonald, Gonasagrie Nair, Camilla Wattrus, Saiqa Mullick, Melanie Pleaner, Elzette Rousseau, Hasina Subedar, Dvora Joseph-Davey, Johan Hugo, Sinead Delany-Moretlwe, Linda-Gail Bekker

**Affiliations:** 1 The Desmond Tutu HIV Centre, Institute of Infectious Disease and Molecular Medicine, Faculty of Health Sciences, University of Cape Town, Cape Town, South Africa; 2 Southern African HIV Clinicians Society (SAHCS), Johannesburg, South Africa; 3 Wits RHI, Faculty of Health Sciences, University of the Witwatersrand, Johannesburg, South Africa; 4 National Department of Health, Pretoria, South Africa; 5 Division of Infectious Diseases, University of California Los Angeles, Los Angeles, United States of America; 6 Department of Epidemiology and Biostatistics, University of Cape Town, Cape Town, South Africa; 7 Anova Health Institute, Cape Town, South Africa

## Introduction

This guideline is an update to the Southern African HIV Clinicians Society (SAHCS) 2020 recommendations on HIV pre-exposure prophylaxis (PrEP).^1^ Previous SAHCS guidance was limited to the provision of oral emtricitabine/tenofovir disoproxil fumarate (F/TDF) PrEP. This update includes newer PrEP modalities, including the monthly dapivirine vaginal ring (DVR) and the long-acting cabotegravir intramuscular injection (CAB-LA). It also introduces upcoming modalities such as the long-acting lenacapavir subcutaneous injection (LEN).

## Background

Significant progress has been made to control the HIV epidemic in South Africa. Since 2010, AIDS-related deaths have decreased by 66%, and new HIV infections have decreased by 58%.^2^ Despite this progress, about 150 000 new HIV cases were reported in South Africa in 2023, which equates to 410 new infections per day.^2^ With no imminent cure, prevention remains the key to epidemic control, hence the significant role of PrEP.

Since 2016, when oral PrEP was initially made available to select sites in South Africa, the provision of PrEP has been scaled up and, as of December 2024, 1 780 919 individuals were initiated on oral PrEP across 4291 facilities.^3,4^ The DVR and CAB-LA were approved for use by the South African Health Products Regulatory Authority (SAHPRA) in 2022, and the South African National Department of Health (NDoH) released guidelines for both products shortly thereafter.^5,6^ However, as of April 2025, they have not been included in the South African Essential Medicines List, are not yet considered standard of care in the public sector, and implementation is limited to a few select study and pilot sites. In the private sector, access to the DVR is steadily increasing, but the introduction of CAB-LA is likely to depend on demand, cost, and willingness of medical aids to reimburse. At present, widescale use of CAB-LA in the private sector may only occur once generic equivalents are approved.

Expanding access to PrEP and newer PrEP modalities, particularly for individuals who are disproportionately affected by and at higher risk of acquiring HIV (including pregnant and lactating people [PLP], people who engage in sex work, transgender and gender diverse [TGD] people, adolescent girls and young women [AGYW], people who use drugs [PWUD] including people who inject drugs [PWID], and gay, bisexual and other men who have sex with men [GBMSM]), could help reduce the number of new HIV infections.

This guideline aims to increase accessibility to PrEP within the South African context. Key updates are detailed in Box 1.

BOX 1 Key updates to the Southern African HIV Clinicians Society HIV pre-exposure prophylaxis guidance.

**Key updates:**

- Expanded eligibility for all PrEP options: PrEP should be offered to all HIV-negative individuals who are at risk of acquiring HIV, as well as to anyone who requests it, independent of risk.
- Increased choice of PrEP products: Addition of the DVR and CAB-LA as PrEP options and information about upcoming modalities, such as lenacapavir.
- Expanded eligibility for intermittent use of PrEP: Eligibility now includes all individuals assigned male at birth who are not on hormone therapy.
- PrEP products for pregnant and lactating people (PLP): Addition of CAB-LA as an option for PLP after risk-benefit assessment.
- Addition of HIV self-testing (HIVST): This is as an option for initiation and maintenance on oral PrEP and the DVR (but not for CAB-LA).
- Increased accessibility to PrEP: A differentiated and simplified approach is recommended.
- Integration with other services: PrEP should be offered as part of an integrated HIV prevention and sexual and reproductive health (SRH) package.
- Guidance on the transition from post-exposure prophylaxis (PEP) to PrEP, and PrEP to PEP.
- PrEP is not a lifelong commitment: Individuals may choose to start, stop and restart PrEP throughout their lives, depending on their lifestyle and requirements.
- Guidance on switching between PrEP products: This depends on the individual’s preferences, circumstances and availability of PrEP products.
- Inclusion of a suggested approach to comprehensive PrEP-choice counselling.
- Updated recommendations for safety screening and monitoring.

PrEP, pre-exposure prophylaxis; DVR, dapivirine vaginal ring; CAB-LA, long-acting cabotegravir intramuscular injection.

The NDoH PrEP guideline^7^ provides valuable guidance for the South African public sector; however, in settings with greater resources and more PrEP options, this guideline provides additional information to enhance the implementation of HIV prevention modalities.

## Initiating pre-exposure prophylaxis

**FIGURE 1:**
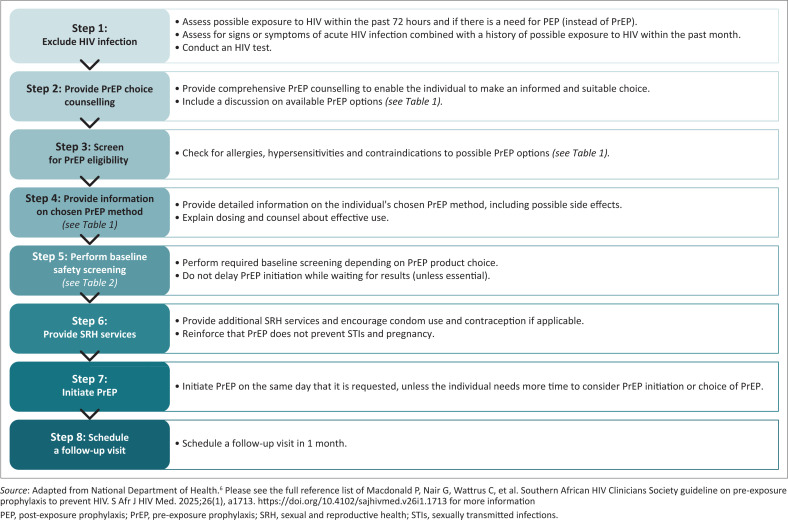
Steps to follow when initiating HIV pre-exposure prophylaxis.

PrEP should be offered as part of an integrated sexual and reproductive health (SRH) package that includes options for HIV prevention to all individuals with a negative HIV test result, and who are likely to be exposed to HIV or who request PrEP. Figure 1 details how to initiate PrEP.

## Selecting an HIV testing strategy

**FIGURE 2:**
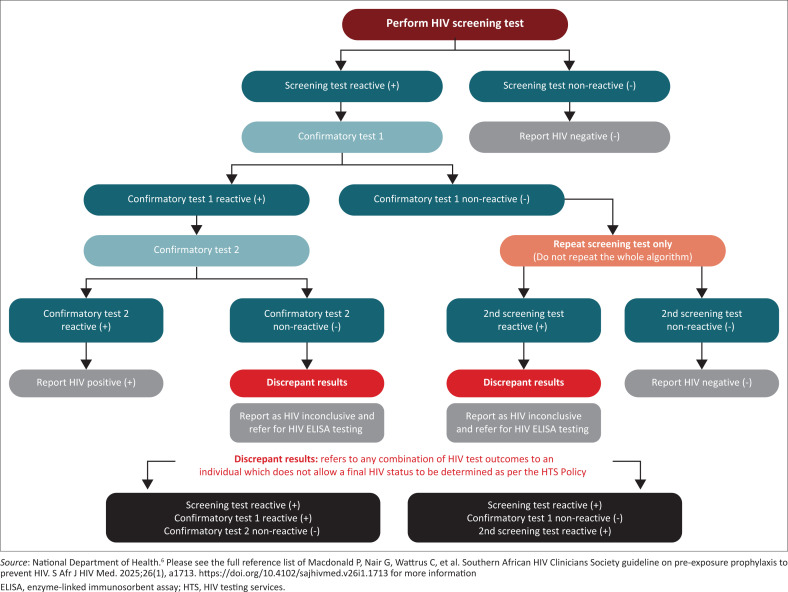
South African National Department of Health HIV testing algorithm.

HIV testing recommendations in the context of PrEP include the following:

- HIV testing is one part of the recommended three-step process to exclude HIV infection (Figure 1, Step 1).
- Test at initiation, 1 month after initiation, and at each follow-up visit thereafter, before PrEP is provided – every 3 months for oral PrEP and DVR, and every 2 months for CAB-LA.
- Follow the NDoH HIV testing algorithm (Figure 2) which recommends three tests to ensure the reliability of positive HIV rapid diagnostic test (RDT) results – a screening test, confirmatory test 1 and confirmatory test 2, with three different HIV assays. This will ensure that a 99% positive predictive value is maintained and aid in avoiding false-positive diagnoses.
- Either a third-generation (HIV antibody) and/or a fourth-generation (HIV antigen/antibody) pre-qualified HIV RDT can be used.
- Individuals with inconclusive RDT results should receive confirmatory testing with a lab-based HIV enzyme-linked immunosorbent assay.
- If resources allow and to reduce the risk of initiating CAB-LA during acute HIV infection, before HIV antibodies can be detected, an HIV nucleic acid antigen test (NAAT) or HIV RNA-1 viral load test can be considered. NAAT assays prior to the initiation of CAB-LA can help prevent the risk of integrase strand transfer inhibitor (INSTI) resistance associated with undiagnosed HIV infection in the context of CAB-LA exposure. If point-of-care NAAT testing is not available, the risk of delaying PrEP initiation should be weighed against the risk of HIV acquisition, while waiting for the result. The impact of these resistance mutations on the effectiveness of the current first-line antiretroviral therapy (ART) treatment regimen of tenofovir/lamivudine/dolutegravir (TLD) is currently unknown.
- If resources allow, NAAT assays can also be considered during follow-up on CAB-LA, in addition to an HIV RDT, to help detect HIV within the context of the Long-acting Early Viral Inhibition (LEVI) syndrome. It is worth noting that, in a recent analysis from HPTN083,^8^ false-positive results have been uncommonly reported, suggesting that NAAT results should be used with caution.
- HIV self-testing (HIVST) may be used instead of provider testing when initiating or continuing oral PrEP or the DVR, but is not currently recommended for use with CAB-LA. In the event of a positive test result obtained through HIVST, this should be confirmed by a trained healthcare provider as soon as possible.

## Pre-exposure prophylaxis choice counselling

Comprehensive counselling on PrEP choice is essential when multiple PrEP products and modalities are available.

**FIGURE 3:**
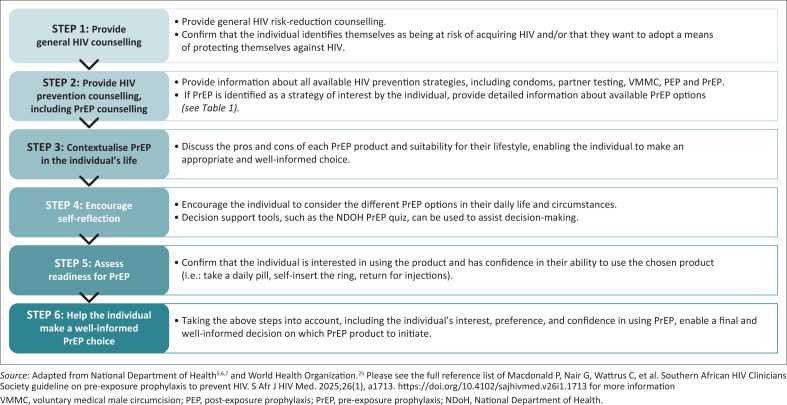
How to provide comprehensive choice counselling for pre-exposure prophylaxis.

Choice counselling should ideally be conducted by a trained healthcare provider, after HIV counselling and testing has been performed, and HIV-negative status confirmed. Counselling should always be person centred, considering the user’s personal preferences alongside eligibility for one or other choice. Figure 3 details a suggested process. Box 2 provides general messages on PrEP choice counselling, and Box 3 provides more information on PWUD.

BOX 2 General messages on pre-exposure prophylaxis choice counselling.

**General messages:**

- PrEP should be framed as a positive, responsible choice that is part of a comprehensive package of HIV prevention and SRH services.
- Individuals should be supported in making their own decisions regarding PrEP choice.
- Additional guidance may be needed during pregnancy and lactation.
- PrEP does not need to be a lifelong commitment.
- PrEP methods can be switched to accommodate lifestyle changes and the individual’s level of risk.

PrEP, pre-exposure prophylaxis; SRH, sexual and reproductive health.

BOX 3 A note on pre-exposure prophylaxis for people who use drugs, including those who inject drugs.

**Notes:**

- There is very limited evidence on PrEP use in PWUD^9^ and currently no efficacy data for CAB-LA and DVR in this group.
- It is important to remember that PWID can also acquire HIV through sex, including particularly high-risk groups such as GBMSM who engage in ChemSex.^10^
- PrEP should be offered as part of a comprehensive harm reduction programme. See here the SAHCS Guidelines for Harm Reduction^11^ for further information.

Note: Chemsex refers to the use of stimulant drugs in sexualised settings to enhance the sexual experience among gay, bisexual, and other men who have sex with men. Also known as party and play (PnP), and slam sex (when in the context of injecting drugs)^10^ Please see the full reference list of Macdonald P, Nair G, Wattrus C, et al. Southern African HIV Clinicians Society guideline on pre-exposure prophylaxis to prevent HIV. S Afr J HIV Med. 2025;26(1), a1713. https://doi.org/10.4102/sajhivmed.v26i1.1713 for more information

PrEP, pre-exposure prophylaxis; PWUD, people who use drugs; PWID, people who inject drugs; GBMSM, gay, bisexual and other men who have sex with men; DVR, dapivirine vaginal ring; CAB-LA, long-acting cabotegravir intramuscular injection; SAHCS, Southern African HIV Clinicians Society.

Individuals who are pregnant, lactating or planning to conceive, and who are at risk of HIV exposure should be encouraged to consider PrEP because (1) there is a higher infection risk, especially in the third trimester and postpartum period^12,13^; (2) the benefits of PrEP extend to protecting the infant by protecting the mother; and (3) the risks to the infant are likely to be minimal. Counselling should cover the risks and benefits of using oral PrEP and CAB-LA, depending on their level of risk of HIV exposure, preference and ability to adhere to a daily pill or injection. Please see *Pre-exposure prophylaxis in pregnant and lactating people* for more detail on PLP.

## Currently available pre-exposure prophylaxis options

Table 1 details available PrEP options in the South African setting, and Table 2 details appropriate safety screening and monitoring.

**TABLE 1:** Approved pre-exposure prophylaxis products in South Africa.

	Oral PrEP		DVR	CAB-LA
	Daily	Intermittent dosing for a single event		
Active ingredients	Each tablet contains TDF 300 mg + FTC 200 mg	-	Silicone vaginal ring containing 25 mg dapivirine	Cabotegravir (600 mg/3 mL) injectable suspension
ARV class	NRTI	-	NNRTI	INSTI
Indications	Anyone with weight ≥ 30 kg Includes adolescents, men, women, TGD individuals, PLP, PWID	Assigned male at birth and ≥ 30 kg and not on gender-affirming hormones Only for sexual exposure, does not protect against HIV during drug injection	Assigned female at birth and ≥ 18 years old Only for vaginal sex, does not protect against HIV exposure during anal sex or drug injection	Anyone with weight ≥ 35 kg Includes adolescents, men, women, TGD individuals, PLP, PWID
Contra-indications	Unknown or positive HIV status Allergy or hypersensitivity to TDF or FTC < 30 kg eGFR < 50 mL/min if > 16 years eGFR < 80 mL/min if 10–16 years If pregnant: serum creatinine > 85 μmol/L	Unknown or positive HIV status Allergy or hypersensitivity to TDF or FTC < 30 kg eGFR < 50 mL/min if > 16 years eGFR < 80 mL/min if 10–16 years On gender-affirming hormones	Unknown or positive HIV status Allergy or hypersensitivity to dapivirine < 18 years old Vaginal ulceration or severe discharge (rather delay use until resolved)	Unknown or positive HIV status Allergy or hypersensitivity to INSTIs < 35 kg Acute hepatitis Advanced liver disease Anticonvulsants – carbamazepine, oxcarbazepine, phenobarbital, phenytoin TB medications – rifampicin, rifapentine
Pregnant and lactating people	Safe and recommended by WHO	Not recommended	Evidence suggests it is safe^17,18^ Awaiting decision from EMA and SAHPRA	Can be used after risk-benefit counselling No increased risk has been demonstrated Recommended by WHO and not contraindicated by SAHPRA Pregnancy outcome monitoring recommended by WHO
Efficacy (when taken as prescribed)	Between 90% and 99%^19^	-	Moderate efficacy: dapivirine release results in a 31% risk reduction in HIV acquisition^20^ and a 63% per-sex-act risk reduction.^21^ Use with condom for additional protection against HIV.	At least 90% effective (possibly as high as 100%)^22,23^
Dosing regimen	Take 1 tablet daily	2-1-1 method: ■ Take 2 tablets 2 h – 24 h before exposure ■ Continue 1 tablet daily during exposure ■ Take 1 tablet daily for 2 days after last exposure	Inserted into vagina and kept in place continuously for 28 days Replace ring every 28 days Do not remove during menstruation or sex	IM gluteal injection given by healthcare provider **Initiation injections:** (2 injections, 1 month apart) ■ Give 1st injection at initiation ■ Give 2nd injection 1 month later **Continuation injections:** ■ Give 2-monthly follow-up injections A 7-day window is permitted before or after the injection due date
Time until protection against HIV	7 days	2 h – 24 h	24 h	7 days
How to safely stop	Continue 1 tablet daily until 7 days after last possible exposure	Take 1 tablet daily for 2 days after last exposure	No protection once removed For ongoing risk of HIV exposure, start alternative method as soon as removed	Provides protection for 8 weeks after last injection Thereafter, CAB level decreases, reducing protection against HIV, and increasing possibility of resistance to ART if HIV infection occurs If ongoing exposure to HIV, use alternative PrEP or HIV prevention method for 12 months after stopping CAB-LA Recommend an HIV test 3-monthly during this tail period
Side effects	Usually mild and resolve after 2–4 weeks Gastrointestinal (nausea, decreased appetite, abdominal cramping, diarrhoea and flatulence), headache, dizziness People with hepatitis B who stop oral PrEP may have sudden worsening of hepatitis symptoms	Usually mild Gastrointestinal (nausea, decreased appetite, abdominal cramping, diarrhoea and flatulence), headache, dizziness	Usually mild Experienced by few users Inflammation of the vagina/vulva/cervix, vaginal discharge/itching, UTIs, pelvic or lower abdominal pain	Usually mild to moderate Experienced by few users Injection site reaction (pain, redness, swelling), headache, nausea, diarrhoea, tiredness
Drug-Drug interactions	No interactions with commonly used medications or hormonal contraceptives Possible additive nephrotoxicity with aminoglycosides (e.g. amikacin and gentamicin for drug-resistant TB)	No interactions with commonly used medications or hormonal contraceptives Possible additive nephrotoxicity with aminoglycosides (e.g. amikacin and gentamicin for drug-resistant TB)	Very low risk as low systemic absorption Avoid/use with caution if concurrent use of other vaginally administered products	Anticonvulsants: carbamazepine, oxcarbazepine, phenobarbital, phenytoin (decrease CAB concentration) Antimycobacterials: rifampicin, rifapentine, rifabutin (decrease CAB concentration) No known interactions with hormonal contraceptives^24^ or gender-affirming hormones

PrEP, Pre-exposure prophylaxis; DVR, dapivirine vaginal ring; CAB-LA, long-acting cabotegravir intramuscular injection; TDF, tenofovir disoproxil fumarate; FTC, emtricitabine; ARV, antiretroviral; NRTI, nucleoside reverse transcriptase inhibitor, NNRTI, non-nucleoside reverse transcriptase inhibitor; INSTI, integrase strand transfer inhibitor, TGD, transgender and gender diverse people; PWID, people who inject drugs; PLP, pregnant and lactating people; eGFR, estimated glomerular filtration rate; IM, intramuscular; CAB, cabotegravir; max, maximum; N/A, not applicable; UTIs, urinary tract infections; EMA, European Medicines Agency; SAHPRA, South African Health Products Regulatory Authority; TB, tuberculosis.

**TABLE 2:** Safety screening and monitoring.

Screening	PrEP option	Who	At PrEP initiation	At PrEP follow-up	Note
HIV infection	Oral PrEP (TDF/FTC) CAB-LA DVR	All	Essential	Essential **CAB-LA**: 1 month after initiation, and then every 2 months **Oral PrEP and DVR:** 1 month after initiation, and then every 3 months	Use point-of-care HIV Ab and/or HIV Ab/Ag RDTs HIVST can be used for Oral PrEP and DVR but not for CAB-LA Consider NAAT testing if available, when initiating CAB-LA
Renal function	Oral TDF-based PrEP only	Well individual, ≤ 30 years	Recommended (but not essential)	Not required	Avoid oral TDF-based PrEP if: ■ eGFR < 50 mL/min if > 16 years ■ eGFR < 80 mL/min if 10–16 years. PrEP can be started while awaiting results As kidney function may vary, day-to-day, eGFR can be repeated to re-assess eligibility
		Well individual, > 30 years	Recommended	6–12 monthly	
		Individuals with comorbidities (hypertension and diabetes)	Recommended	6-monthly	
		Concomitant nephrotoxic medication	Recommended	6-monthly	
		Pregnant	Recommended	3 and 6 months after initiation (during pregnancy)	Avoid oral TDF-based PrEP if serum creatinine > 85 μmol/L. PrEP can be started while awaiting results. As kidney function may vary, day-to-day, eGFR can be repeated to re-assess eligibility
Liver function (ALT and AST)	CAB-LA	All	Optional unless concomitant hepatitis B (then recommended)	Not required	Consider if suspected hepatotoxicity (e.g. high alcohol use)
Hepatitis B (HbsAg)	Oral PrEP CAB-LA	All	Recommended Testing availability should not be a barrier to PrEP initiation	Not required	CAB-LA provides no benefit in hepatitis B infection, unlike TDF which can be used to treat hepatitis B and may be preferred PrEP can be started while awaiting results If HbsAg positive, consider specialist referral
Hepatitis C	Oral PrEP CAB-LA	All	Recommended Testing availability should not be a barrier to PrEP initiation	Not essential	CAB-LA provides no benefit in hepatitis C infection If hepatitis C positive, rather consider oral TDF-based PrEP and refer for DAA treatment PrEP can be started while awaiting results
STI screen, (Including *Chlamydia trachomatis, Neisseria gonorrhoea*, syphilis)	Oral PrEP (TDF/FTC) CAB-LA DVR	All	Recommended	Symptom screen 3 monthly If available, STI testing 6 monthly	STI diagnostic testing is recommended even if asymptomatic If not available and symptomatic, manage syndromically
Pregnancy	Oral PrEP (TDF/FTC) CAB-LA DVR	Females of reproductive age	Recommended, if applicable	Recommended, if applicable	Rapid pregnancy tests can be used. If pregnant, PrEP options include oral PrEP and CAB-LA following risk-benefit counselling.

*Source*: Adapted from National Department of Health^5,6,7^ and World Health Organization.^19^ Please see the full reference list of Macdonald P, Nair G, Wattrus C, et al. Southern African HIV Clinicians Society guideline on pre-exposure prophylaxis to prevent HIV. S Afr J HIV Med. 2025;26(1), a1713. https://doi.org/10.4102/sajhivmed.v26i1.1713 for more information

PrEP, Pre-Exposure Prophylaxis; DVR, dapivirine vaginal ring; CAB-LA, long-acting cabotegravir intramuscular injection; TDF, tenofovir disoproxil fumarate; FTC, emtricitabine; eGFR, estimated glomerular filtration rate; IM, intramuscular; CAB, cabotegravir; STI, sexually transmitted infection; RDT, rapid diagnostic test; AST, aspartate transaminase; ALT, alanine transaminase; HBsAg, Hepatitis B surface antigen; DAA, direct-acting antiviral; HIVST, HIV self-test; NAAT, nucleic acid amplification test; Ab, antibody; Ag, antigen; STI, sexually transmitted infection.

## Integrated HIV prevention and sexual and reproductive health package

PrEP should be offered as part of an integrated HIV prevention and SRH package to all individuals with a negative HIV test, and who are likely to be exposed to HIV or who request PrEP. Box 4 outlines the recommended core services, along with additional services to consider or for which to establish referral pathways.

BOX 4 Components of an integrated HIV prevention and sexual and reproductive health package.

**Core services:**

- HIV counselling and testing, including partner testing.
- Risk reduction counselling.
- Provision of condoms (male and female) and lubricant.
- Provision of PEP (if indicated).
- PrEP choice counselling.
- Provision of PrEP.
- STI screening and treatment (including partner notification[s]):
  ■ Ideally aetiological testing: *Chlamydia trachomatis, Neisseria gonorrhoea, Treponema pallidum* (syphilis), *Trichomonas vaginalis.*
  ■ STI syndromic management if STI testing unavailable.
- Hepatitis B and C screening.
- Pregnancy screening in women of reproductive age.
- Provision of contraception.
- TB screening (and partner screening/notification when applicable).
- Mental health screening, counselling and referral when necessary.
- Gender-based violence and intimate partner violence assessment and response.

**Additional services to consider / referrals:**

- General primary healthcare services (when applicable).
- Antenatal care.
- Termination of pregnancy.
- Antiretroviral treatment for individuals and their partner(s) (when applicable).
- Gender-affirming care services (when applicable).
- Harm reduction services for people who use drugs.
- Prevention, assessment and treatment of cervical cancer.
- HPV vaccination.

*Source*: Adapted from National Department of Health^7^ and World Health Organization.^19^ Please see the full reference list of Macdonald P, Nair G, Wattrus C, et al. Southern African HIV Clinicians Society guideline on pre-exposure prophylaxis to prevent HIV. S Afr J HIV Med. 2025;26(1), a1713. https://doi.org/10.4102/sajhivmed.v26i1.1713 for more information

PrEP, pre-exposure prophylaxis; PEP, post-exposure prophylaxis; STI, sexually transmitted infection; TB, tuberculosis; HPV, human papilloma virus.

## Differentiated service delivery

To improve PrEP uptake and persistence within an integrated HIV prevention and SRH service, and recognising that PrEP users are generally healthy, a **differentiated service delivery** model has been proposed.^14^ This model leverages existing platforms and introduces alternative delivery options outside traditional public health services, including private practice clinicians, mobile clinics, pharmacies, schools, and courier services. **Task shifting** of PrEP provision from doctors to nurses, and other trained and authorised providers such as pharmacists and counsellors, is encouraged. The **simplification** of PrEP provision aims to make access easier while maintaining quality of care. One example is multi-month dispensing, which allows for the provision of up to 6 months’ supply of oral PrEP or the DVR at follow-up visits, combined with HIVST kits. This approach reduces the need for frequent visits to a healthcare facility or a clinician, streamlining access to the service.^14,15,16^

## Follow-up visits

**FIGURE 4:**
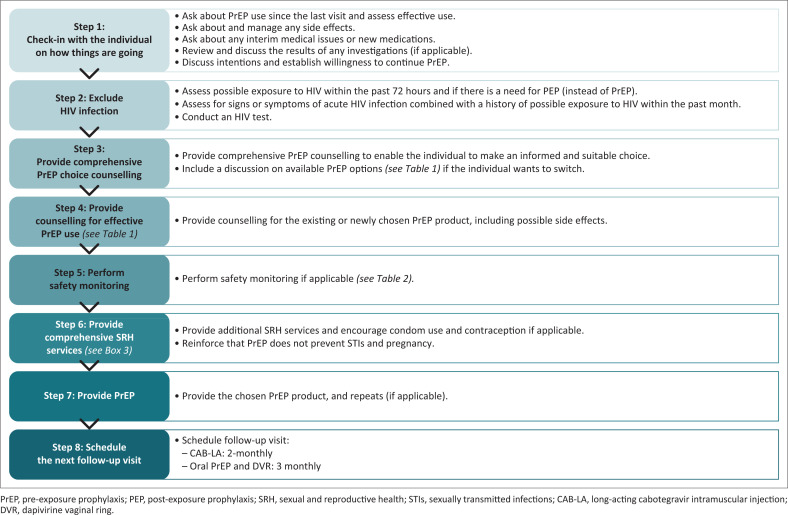
HIV pre-exposure prophylaxis follow-up visits.

Figure 4 details what should be done at PrEP follow-up visits.

## Cycling on and off pre-exposure prophylaxis

It is important that individuals who choose to start, stop and restart PrEP do so safely, and that they are well educated about:

- The lead-in time until protection against HIV, when starting or restarting PrEP.
- How to safely stop the PrEP method after their last potential exposure, and the need to use alternative HIV prevention methods, if necessary.

Cycling on and off CAB-LA intermittently is not recommended, because of the long tail, and the need to cover with other HIV prevention methods (usually oral PrEP), to prevent INSTI resistance if HIV infection occurs during the period of subtherapeutic tissue levels.

## Switching between pre-exposure prophylaxis products

Individuals may choose to switch between PrEP products, depending on their preferences, changing circumstances, and the availability of certain PrEP products. If there is ongoing potential exposure to HIV, providers should support an individual to choose an alternative PrEP method and support them to switch correctly and safely to maintain optimal protection against HIV.

Both the lead-in time to effectiveness of the new product, and the waning effectiveness of the current product, need to be considered when switching. Simultaneous use of the two products for a limited period, to cover their lead-in and tail periods, may be considered.^19^ For example, an individual may be on both CAB-LA and oral PrEP for 7 days when stopping oral PrEP and starting CAB-LA. There are limited data on the safety of using more than one PrEP product at once, but no serious concerns are anticipated.

Table 3 details how to safely switch between PrEP products.

**TABLE 3:** Switching between pre-exposure prophylaxis products.

Switch	Note
Oral PrEP or DVR to CAB-LA	Use oral PrEP or DVR for 7 days after starting CAB-LA.
CAB-LA to Oral PrEP or DVR	Start oral PrEP or DVR 8 weeks after the last CAB-LA injection (less than 8 weeks can be considered if it facilitates engagement in care and retention). CAB-LA provides protection for 8 weeks after the last injection. Thereafter, the level of CAB decreases, reducing the level of protection against HIV, and increasing the possibility of resistance to ART if HIV infection occurs, for up to 12 months after stopping CAB-LA. An alternative HIV prevention method should be used for any ongoing possible HIV exposure for up to 12 months after stopping CAB-LA.
Oral PrEP to DVR	Use oral PrEP for 7 days after DVR insertion.
DVR to Oral PrEP	Use the DVR (or alternative HIV prevention, such as condoms) for 7 days after starting oral PrEP.

*Source*: World Health Organization.^19^ Please see the full reference list of Macdonald P, Nair G, Wattrus C, et al. Southern African HIV Clinicians Society guideline on pre-exposure prophylaxis to prevent HIV. S Afr J HIV Med. 2025;26(1), a1713. https://doi.org/10.4102/sajhivmed.v26i1.1713 for more information

Note: The ideal strategy for transitioning between different PrEP products, including overlapping use of PrEP products, is not currently known. There is also limited data on the safety of using more than one PrEP product at a time. There is no evidence suggesting that using multiple PrEP products at the same time results in any advantage in terms of reduced risk of HIV acquisition (beyond the advantages of each PrEP product individually).

PrEP, pre-exposure prophylaxis; DVR, dapivirine vaginal ring; CAB-LA, long-acting cabotegravir intramuscular injection; CAB, cabotegravir; ART, antiretroviral therapy.

## Post-exposure prophylaxis to pre-exposure prophylaxis (and pre-exposure prophylaxis to post-exposure prophylaxis) transitions

PEP is the administration of a short course of ART to an individual who has an HIV-negative test and who may have been exposed to HIV, in order to prevent acquisition of the virus. PEP can effectively prevent infection in a person exposed to HIV when initiated as soon as possible, ideally within 24 h, and at least within 72 h post-exposure. The global recommendation for PEP is a three-drug regimen involving, whenever possible, an integrase inhibitor (usually dolutegravir).^25^ Table 4 details how to safely transition between PEP and PrEP.

**TABLE 4:** Transitioning from post-exposure prophylaxis to pre-exposure prophylaxis and pre-exposure prophylaxis to post-exposure prophylaxis.

Transition	Note
**PEP to PrEP** (Oral PrEP, DVR or CAB-LA)	Start oral PrEP/DVR/CAB-LA immediately after completing the 28-day course of PEP and *after confirming a negative HIV result*. PrEP initiation following PEP can be managed the same as anyone else starting PrEP. Immediate transition from PEP to PrEP is recommended for individuals who have an ongoing risk of HIV exposure.
**PrEP to PEP**	PEP is not usually required if PrEP is used as prescribed; but if there are concerns about adherence it may be safer to pause PrEP and start PEP instead to ensure protection. Providers should educate all PrEP users about PEP, and emergency STI prophylaxis and contraception should the need arise.

*Source*: World Health Organization.^19^ Please see the full reference list of Macdonald P, Nair G, Wattrus C, et al. Southern African HIV Clinicians Society guideline on pre-exposure prophylaxis to prevent HIV. S Afr J HIV Med. 2025;26(1), a1713. https://doi.org/10.4102/sajhivmed.v26i1.1713 for more information

PrEP, pre-exposure prophylaxis; PEP, post-exposure prophylaxis; DVR, dapivirine vaginal ring; CAB-LA, long-acting cabotegravir intramuscular injection; STI, sexually transmitted infection.

## Managing missed pre-exposure prophylaxis doses

There are many reasons why PrEP users may miss a dose or more, and PrEP providers should be supportive and non-judgemental when this happens. This is often a good time to discuss different PrEP options, choices and the effective use of PrEP, within an integrated HIV prevention and SRH context. Table 5 details how to manage a missed PrEP dose.

**TABLE 5:** Managing missed pre-exposure prophylaxis doses.

Modality	Note
**Oral PrEP** (Daily or intermittent)	If 1 dose is missed, take 1 tablet as soon as remembered. If > 2 days without dose, restart oral PrEP as prescribed. Counsel for additional protection, if required.
**DVR**	If expelled/removed and no exposure to unhygienic environment, rinse ring in clean water and immediately reinsert. Alternatively, replace with new ring. Counsel for additional protection, if required.
**CAB-LA**	Dosing schedule after *unplanned* missed injections: *How much time has passed since the missed target injection date?* **If ≤ 4 weeks since target injection date:** ■ Resume injections as soon as possible. ■ Continue with every-2-month dosing schedule thereafter. **If ≥ 4 weeks since target injection date:** ■ Repeat initiation injections (2 injections 1 month apart). ■ Continue with every-2-month dosing schedule thereafter. Oral PrEP bridging for *planned* missed injections: If an individual plans to miss their target injection date by > 7 days, daily oral prep can be prescribed to bridge the gap between injections. Start oral PrEP on the target injection date and continue daily until able to return. When the individual returns, apply the same dosing schedule as above: *How m uch time has passed since the missed CAB-LA target injection date?* **If ≤ 4 weeks since target injection date:** ■ Resume injections as soon as possible. ■ Continue with every-2-month dosing schedule thereafter. **If ≥ 4 weeks since target injection date:** ■ Repeat initiation injections (2 injections 1 month apart). ■ Continue with every-2-month dosing schedule thereafter.

*Source*: Adapted from National Department of Health^7^ and World Health Organization.^19^ Please see the full reference list of Macdonald P, Nair G, Wattrus C, et al. Southern African HIV Clinicians Society guideline on pre-exposure prophylaxis to prevent HIV. S Afr J HIV Med. 2025;26(1), a1713. https://doi.org/10.4102/sajhivmed.v26i1.1713 for more information

PrEP, pre-exposure prophylaxis; DVR, dapivirine vaginal ring; CAB-LA, long-acting cabotegravir intramuscular injection.

## Managing HIV seroconversion

HIV seroconversion while on PrEP is very unlikely if it is used effectively, but can occur if:

- PrEP is initiated in an individual with pre-existing HIV infection that is missed.
- PrEP is not used effectively, and HIV exposure occurs after starting PrEP (break through infections).

If HIV seroconversion occurs, PrEP should be stopped and ART started immediately, or as soon as possible after the diagnosis of HIV is confirmed.

If the individual is using oral PrEP (daily or intermittently) or the DVR before HIV seroconversion, first-line ART (TLD) can be started, as the risk of developing HIV drug resistance with oral PrEP and the DVR is low.

If the individual is using CAB-LA, there is a possibility that drug resistance may develop to dolutegravir as both cabotegravir and dolutegravir are INSTIs. There are three possible scenarios to consider when HIV seroconversion occurs in the context of CAB-LA use:

- Undiagnosed HIV infection at CAB-LA initiation.
- Breakthrough HIV infection while on CAB-LA.
- HIV infection acquired after stopping CAB-LA, while levels of CAB-LA are waning in the 12-month tail period.

The diagnosis of HIV might be delayed due to the suppression and inhibition of the HIV virus by CAB-LA, resulting in false-negative HIV tests. This is known as the LEVI syndrome, and if ongoing CAB-LA injections continue (as HIV infection is masked), then the monotherapy may result in the development of INSTI resistance.^26^

When HIV seroconversion occurs in the context of CAB-LA in any of the above three scenarios, TLD should be started immediately, and an HIV drug-resistance test done. Consultation with an experienced clinician is also recommended.

## Pre-exposure prophylaxis in pregnant and lactating people

PrEP is recommended for all PLP who are at risk of HIV exposure, because pregnancy is a time when the risk of HIV infection and transmission to the infant is higher, with the risk more than doubling during pregnancy and the postpartum period.^12,13^

Women who acquire HIV during pregnancy or lactation have a higher chance of transmitting HIV to their infants. Consequently, primary HIV prevention for PLP has emerged as a key global health and South African HIV prevention priority.

As of April 2025, PrEP options for PLP in South Africa include daily oral PrEP and CAB-LA following risk-benefit counselling. The DVR is not approved for use in PLP, although evidence suggests that it is safe to use in pregnancy, and SAHPRA approval is pending.

**Oral PrEP:** Studies have shown that exposure to TDF and FTC during pregnancy among women living with HIV is safe and well tolerated. A recent systematic review^27^ identified 14 studies that evaluated or will evaluate maternal and/or infant outcomes following oral PrEP exposure during pregnancy or lactation. None of the completed studies found differences in pregnancy or perinatal outcomes associated with oral PrEP exposure. Most pregnant people do not experience significant side effects from oral PrEP; however, about one in 10 may have side effects such as nausea, abdominal cramps, or headache. These are usually mild and resolve in the first few weeks. Some side effects of oral PrEP may also be confused with symptoms of early pregnancy (e.g. nausea, vomiting, and fatigue).

**CAB-LA** is well tolerated in pregnant women. Maternal, pregnancy, and immediate infant outcomes (as defined by WHO recommended endpoints) are comparable to F/TDF, with expected background rates. Pharmacokinetic (PK) analyses showed a decline in concentrations, particularly in the third trimester, although these declines were above target thresholds, and no HIV infections occurred during pregnancy.^28,29^ Currently, no dose adjustment is required; however, analyses are ongoing to confirm this. Regarding infant outcomes, the use of CAB-LA has not been associated with an increase in spontaneous abortions or congenital abnormalities.^28,29,30^ Details regarding CAB exposure in infants who breastfeed is unknown. It is expected that transmission through breastmilk is minimal and similar to levels seen for dolutegravir, but this will be confirmed in ongoing studies.

The **DVR** has not yet been approved by SAHPRA for use in PLP; however, there is now data to indicate its safety for use in both pregnant and lactating people,^17,18^ with pregnancy and infant outcomes being similar to or lower than local background rates.^18,27,31^ Dapivirine exposure to infants during breastfeeding is safe, and the quantity of dapivirine in breastmilk from a DVR is minimal.^29,32,33^

When choosing PrEP during pregnancy and lactation, it is important to consider all potential risks and benefits, making decisions on a case-by-case basis. Common (and temporary) side effects, such as nausea and fatigue, which can occur in both early pregnancy and early PrEP use, should be discussed during counselling, and reassurance provided to encourage PrEP use and support ongoing adherence.

## Upcoming modalities

The HIV prevention pipeline has many products in pre-clinical and clinical development, including preventive vaccines, broadly neutralising antibodies (BNAbs), monthly oral pills, vaginal rings, vaginal and rectal gels, vaginal films, long-acting injectable antiretrovirals, and multi-purpose prevention technologies (MPTs) designed to reduce the risk of HIV and sexually transmitted infections and/or provide effective contraception for women.^34^

Most relevant, and within reach is lenacapavir (a 6-monthly subcutaneous long-acting PrEP injection), a monthly pill with the nucleoside reverse transcription translocation inhibitor (MK8527), the three-monthly DVR, and a dual prevention pill combining oral PrEP and the oral contraceptive, levonorgestrel.

**Lenacapavir (LEN)** is a first-in-class multi-stage capsid inhibitor that can be administered every six months via subcutaneous injection. The FDA is expected to approve LEN for HIV prevention soon, following the promising results of the PURPOSE 1^35^ and PURPOSE 2^36^ studies. PURPOSE 1 evaluated the efficacy and safety of LEN for HIV prevention in cisgender women from South Africa and Uganda, aged 16-25 years. Among the 2134 cisgender women receiving twice-yearly LEN, none acquired HIV, demonstrating 100% effectiveness. LEN was also safe for women who became pregnant while using it. Participants and infants continue to be followed in the open-label extension phase and will contribute to pharmacokinetic (PK) data.^35^ PURPOSE 2 showed that LEN reduced the risk of acquiring HIV by 96% compared to background HIV incidence among 2183 participants, including cisgender men, transgender men, transgender women, and nonbinary individuals aged 16 years and older.^36^ PURPOSE 1 also assessed the protection against HIV of daily oral tenofovir alafenamide, but this did not differ from background HIV infection rates or the standard oral F/TDF.^35^ A matched control study of drug levels confirmed this was likely due to inadequate adherence by the women in the oral PrEP arms.

**MK8527** is a long-acting nucleoside reverse transcriptase translocation inhibitor which due to its antiretroviral potency can be administered monthly as a small pill. Phase 2 dose finding studies are completed and phase 3 trials will be commencing in 2025. It is hoped efficacy data may be available in 2027.

**A three-monthly DVR** is also in development, and offers several advantages over the monthly DVR, including reductions in cost, waste and replacement frequency, and acceptibility.^37^ Recent data suggest that the efficacy of the 3-month ring will be at least equal to that of the 1-month DVR, and provide an extended-use option.^38,39^

The **dual prevention pill:** This MPT is the first to contain PrEP. An application for regulatory approval of this combination of two already approved products (TDF/FTC) and oral contraception (ethinyl estradiol and levonorgestrel) was made in 2024, and it is anticipated that approvals will be received in late 2025. Bioequivalence studies have confirmed that available concentrations of active drugs in the MPT are the same as for each individual product.^40^

**Tenofovir/levonorgestrel (TFV/LNG) vaginal ring:** A phase 2a study conducted among Kenyan women showed that the TFV/LNG ring was safe and well tolerated. PK studies indicated that clinical efficacy for prevention of HIV-1, HIV-2 infections and pregnancy were highly likely.^41^

**BNAbs** administered as infusions or subcutaneous injections are also being considered as a type of immune-based prophylaxis.^42^ This is based on the proof-of-concept studies that showed that the BNAbs were protective if it matched the circulating HIV.^43^

**Quick-start oral PrEP** for women for intermittent use is also being discussed, and inclusion in guidelines is contingent on the availability of supportive efficacy data. This is also known as the 2:7 method, as opposed to the 2:1:1 method for men. Two tablets are taken 2–24 h before exposure and then 1 tablet is taken every day for 7 days after possible exposure.

## Conclusion

More than 10 years after TDF-based oral PrEP was first approved for use for HIV prevention, two novel long-acting modalities have been approved within a relatively short space of 2 years of each other (DVR and CAB-LA). Six-monthly LEN shows great promise, and regulatory approval is eagerly awaited. Additional long-acting methods and MPTs and devices are set to enter clinical trials, or have shown promise in early clinical development. Several mechanisms have been proposed to increase accessibility and guidelines developed to allow providers to confidently offer and manage individuals who choose PrEP. Advances toward holistic, person-centred, preference-based PrEP provision will bring us closer to realising our goal to end HIV.

## References

[1] Bekker L, Brown B, Joseph-Davey D. Southern African guidelines on the safe, easy and effective use of pre-exposure prophylaxis: 2020. South Afr J HIV Med. 2020;21(1):a1152. 10.4102/sajhivmed.v21i1.1152 PMC7736681 33354364

[2] UNAIDS. South Africa [homepage on the Internet]. 2025 [cited 2025 Mar 31]. Available from: https://www.unaids.org/en/regionscountries/countries/southafrica

[3] TB/HIV Information System (TIER.net). South African National Department of Health; 2025 [cited 2025 Feb 17]. Available from: https://TIER.net.

[4] District Health Information System (DHIS). South African National Department of Health; 2025 [cited 2025 Feb 17]. Available from: https://login.health.gov.za/userman/landwelcome.php

[5] National Department of Health. National Dapivirine Vaginal Ring Implementation Guidelines 02 December Updated. Pretoria: National Department of Health. Republic of South Africa; 2022.

[6] National Department of Health. National Implementation Guidelines for Long Acting Injectable Cabotegravir (CAB-LA). Pretoria: National Department of Health. Republic of South Africa; 2023.

[7] National Department of Health. Updated Guidelines for the provision of Oral Pre-Exposure Prophylaxis (PrEP) to persons at substantial risk of infection. Pretoria: National Department of Health. Republic of South Africa; 2021.

[8] Fogel JM, Piwowar-Manning E, Moser A, et al. Evaluation of Xpert point-of-care assays for detection of HIV infection in persons using long-acting cabotegravir for pre-exposure prophylaxis. Microbiology Spectrum. 2024;12(8):e00307–e00324. 38980027 10.1128/spectrum.00307-24 PMC11302132

[9] Choopanya K, Martin M, Suntharasamai P, et al. Antiretroviral prophylaxis for HIV infection in injecting drug users in Bangkok, Thailand (the Bangkok Tenofovir Study): A randomised, double-blind, placebo-controlled phase 3 trial. Lancet. 2013;381(9883):2083–2090. 10.1016/S0140-6736(13)61127-7 23769234

[10] Strong C, Huang P, Li C-W, Ku SW-W, Wu H-J, Bourne A. HIV, chemsex, and the need for harm-reduction interventions to support gay, bisexual, and other men who have sex with men. The lancet HIV. 2022;9(10):e717–e725. 10.1016/S2352-3018(22)00124-2 35926550

[11] Scheibe A, Goodman Sibeko SS, Rossouw T, Zishiri V, Venter WD. Southern African HIV Clinicians Society guidelines for harm reduction. South Afr J HIV Med. 2020;21(1):1161. 10.4102/sajhivmed.v21i1.1161 33391833 PMC7756663

[12] Graybill LA, Kasaro M, Freeborn K, et al. Incident HIV among pregnant and breast-feeding women in sub-Saharan Africa: A systematic review and meta-analysis. AIDS. 2020;34(5):761–776. 10.1097/QAD.0000000000002487 32167990 PMC7275092

[13] Thomson KA, Hughes J, Baeten JM, et al. Increased risk of HIV acquisition among women throughout pregnancy and during the postpartum period: A prospective per-coital-act analysis among women with HIV-infected partners. J Infect Dis. 2018;218(1):16–25. 10.1093/infdis/jiy113 29514254 PMC5989601

[14] World Health Organization. Differentiated and simplified pre-exposure prophylaxis for HIV prevention: Update to WHO implementation guidance. Technical Brief. Geneva: World Health Organization; 2022.

[15] Green KE, Nguyen LH, Phan HTT, et al. Prepped for PrEP? Acceptability, continuation and adherence among men who have sex with men and transgender women enrolled as part of Vietnam’s first pre-exposure prophylaxis program. Sex Health. 2021;18(1):104–115. 10.1071/SH20167 33653505

[16] Janamnuaysook R, Green KE, Seekaew P, et al. Demedicalisation of HIV interventions to end HIV in the Asia–Pacific. Sex Health. 2021;18(1):13–20. 10.1071/SH20172 33632380

[17] Makanani B, Balkus JE, Jiao Y, et al. Pregnancy and infant outcomes among women using the dapivirine vaginal ring in early pregnancy. J Acquir Immune Defic Syndr. 2018;79(5):566–572. 10.1097/QAI.0000000000001861 30383589 PMC6231990

[18] Bunge K, Balkus JE, Fairlie L, et al. DELIVER: A safety study of a dapivirine vaginal ring and oral PrEP for the prevention of HIV during pregnancy. J Acquir Immune Defic Syndr. 2022;10:1097. 10.1097/QAI.0000000000003312 PMC11443417 38055292

[19] World Health Organization. WHO implementation tool for pre-exposure prophylaxis (PrEP) of HIV infection: Provider module for oral and long-acting PrEP. Geneva: World Health Organization; 2024.

[20] Nel A, Van Niekerk N, Van Baelen B, et al. Safety, adherence, and HIV-1 seroconversion among women using the dapivirine vaginal ring (DREAM): An open-label, extension study. Lancet HIV. 2021;8(2):e77–e86. 10.1016/S2352-3018(20)30300-3 33539761

[21] Stalter RM, Dong TQ, Hendrix CW, et al. Assessing per-sex-act HIV-1 risk reduction among women using the dapivirine vaginal ring. J Infect Dis. 2024;229(4):1158–1165. 10.1093/infdis/jiad550 38099506 PMC11011174

[22] Delany-Moretlwe S, Hughes JP, Bock P, et al. Cabotegravir for the prevention of HIV-1 in women: Results from HPTN 084, a phase 3, randomised clinical trial. Lancet. 2022;399(10337):1779–1789. 10.1016/S0140-6736(22)00538-4 35378077 PMC9077443

[23] Landovitz RJ, Hanscom BS, Clement ME, et al. Efficacy and safety of long-acting cabotegravir compared with daily oral tenofovir disoproxil fumarate plus emtricitabine to prevent HIV infection in cisgender men and transgender women who have sex with men 1 year after study unblinding: A secondary analysis of the phase 2b and 3 HPTN 083 randomised controlled trial. Lancet HIV. 2023;10(12):e767–e778. 10.1016/S2352-3018(23)00261-8 37952550 PMC11375758

[24] Spinelli MA, Grinsztejn B, Landovitz RJ. Promises and challenges: Cabotegravir for preexposure prophylaxis. Curr Opin HIV AIDS. 2022;17(4):186–191. 10.1097/COH.0000000000000733 35762372 PMC9240402

[25] Horak J, Venter WD, Wattrus C, et al. Southern African HIV Clinicians Society 2023 Guideline for post-exposure prophylaxis: Updated recommendations. South Afr J HIV Med. 2023;24(1):1522. 10.4102/sajhivmed.v24i1.1522 37795431 PMC10546897

[26] Landovitz RJ, Delany-Moretlwe S, Fogel JM, et al. Features of HIV infection in long-acting cabotegravir pre-exposure prophylaxis. The New England Journal of Medicine. 2024;391(13):1253. 39046350 10.1056/NEJMc2402088 PMC11598663

[27] Erlwanger A, Rocroi I, Kirtley S, Hemelaar J. Perinatal outcomes associated with pre-exposure prophylaxis for HIV prevention during pregnancy: A systematic review and meta-analysis. Eclinicalmedicine. 2024;70:102532. 10.1016/j.eclinm.2024.102532 38685925 PMC11056414

[28] Delany-Moretlwe S, Hughes J, Guo X, et al. Evaluation of CAB-LA safety and PK in pregnant women in the blinded phase of HPTN 084. Paper presented at: Conference on Retroviruses and Opportunistic Infections (CROI); 2022 Feb 12–16.

[29] Delany-Moretlwe S, Hanscom B, Guo X, et al. Evaluation of long-acting cabotegravir safety and pharmacokinetics in pregnant women in eastern and southern Africa: A secondary analysis of HPTN 084. J Int AIDS Soc. 2025;28(1):e26401. 10.1002/jia2.26401 39748218 PMC11695207

[30] Johnson LF, Myer L, Jamieson L, Meyer-Rath G, Delany-Moretlwe S, Davey DJ. The potential benefits of long-acting injectable cabotegravir in pregnant and breastfeeding women and their infants: A modelling study. AIDS. 2022:10.1097. 10.1097/QAD.0000000000003803 PMC10906189 38016171

[31] Mhlanga F, Bunge K, Fairlie L. Safety of dapivirine vaginal ring and oral PrEP for HIV prevention in the second trimester. Paper presented at: Conference on Retroviruses and Opportunistic Infections; 2024 Mar 3–6; Denver.

[32] Noguchi LM, Hoesley C, Kelly C, et al. Pharmacokinetics of dapivirine transfer into blood plasma, breast milk, and cervicovaginal fluid of lactating women using the dapivirine vaginal ring. Antimicrob Agents Chemother. 2019;63(3). 10.1128/AAC.01930-18 PMC6395928 30602513

[33] Noguchi LM, Owor M, Mgodi NM, et al. Safety and drug quantification of the dapivirine vaginal ring and oral pre-exposure prophylaxis in breastfeeding mother–infant pairs (MTN-043): A phase 3B, open-label, randomised trial. Lancet HIV. 2024. 10.1016/S2352-3018(24)00306-0 PMC12338333 39954697

[34] The HIV prevention pipeline [homepage on the Internet]. [cited 2025 Feb 17]. Available from: https://avac.org/resource/infographic/the-hiv-prevention-pipeline

[35] Bekker L-G, Das M, Abdool Karim Q, et al. Twice-yearly lenacapavir or daily F/TAF for HIV prevention in cisgender women. N Engl J Med. 2024;391(13):1179–1192. 10.1056/NEJMoa2407001 39046157

[36] Kelley CF, Acevedo-Quiñones M, Agwu AL, et al. Twice-Yearly Lenacapavir for HIV Prevention in Men and Gender-Diverse Persons. N Engl J Med. 2024 Nov 27;12(8):e00307–e00324. 10.1056/NEJMoa2407001 39602624

[37] Friedland B, Nunu M, Clarke-Von Witt S, et al. Acceptability of the 3-month dapivirine ring versus the 1-month dapivirine vaginal ring: Qualitative findings from a crossover bioavailability trial among 18–45-year-old women in Bloemfontein, South Africa. Paper presented at: HIVR4P 2024, the 5th HIV Research for Prevention Conference 2024; Lima, Peru.

[38] Relative bioavailability trial of dapivirine ring-004 and ring-008 [homepage on the Internet]. ClinicalTrials.gov; 2024 [cited 2025 Feb 14]. Available from: https://clinicaltrials.gov/study/NCT05416021?term=NCT05416021&rank=1

[39] Nuttall J, Haddad L, Plagianos M, et al. Pharmacokinetic superiority of a 3-month dapivirine vaginal ring (100 mg) compared to the 1-month dapivirine vaginal ring (25 mg). Paper presented at: Journal of the International Aids Society 2024.

[40] Dual prevention pill [homepage on the Internet]. [cited 2025 Feb 17]. Available from: https://www.prepwatch.org/products/dual-prevention-pill/

[41] Mugo NR, Mudhune V, Heffron R, et al. Randomized controlled phase IIa clinical trial of safety, pharmacokinetics and pharmacodynamics of tenofovir and tenofovir plus levonorgestrel releasing intravaginal rings used by women in Kenya. Front Reprod Health. 2023;5:1118030. 10.3389/frph.2023.1118030 37383290 PMC10293630

[42] Fidler S, Caskey M. Potential for broadly neutralising antibodies as PrEP. Lancet HIV. 2025;12(1):e2–e3. 10.1016/S2352-3018(24)00308-4 39667378

[43] Corey L, Gilbert PB, Juraska M, et al. Two randomized trials of neutralizing antibodies to prevent HIV-1 acquisition. N Engl J Med. 2021;384(11):1003–1014. 10.1056/NEJMoa2031738 33730454 PMC8189692

